# Ability of the PulseCO Algorithm to Assess Changes in Stroke Volume Induced by Changes in Vascular Tone

**DOI:** 10.1111/aas.70338

**Published:** 2026-09-27

**Authors:** Jonathan Winther Olsen, Henrik Wolsted, Rune Sort, Mirjana Cihoric, Nicolai Bang Foss, Jakob Højlund

**Affiliations:** ^1^ Department of Anesthesiology and Intensive Care Copenhagen University Hospital–Hvidovre Copenhagen Denmark

## Abstract

**Background:**

Vasoconstriction during anesthesia can influence stroke volume (SV) by preload modulation, making accurate SV monitoring essential to guide vasopressor therapy. Since gold standard methods like pulmonary artery catheterization and lithium dilution provide only intermittent estimates, continuous and less invasive arterial pulse contour analysis systems are increasingly used. However, their accuracy under altered vascular tone remains uncertain. We aimed to evaluate the PulseCO algorithm's (SV‐P) ability to detect clinically relevant changes in SV index (SVI) during manipulation of vascular tone, using lithium dilution with the LiDCO method (CI‐Li) as reference, and to assess how absolute systemic vascular resistance index (SVRI) levels influenced agreement between methods.

**Methods:**

In this prospective observational study, adult patients undergoing general anesthesia were monitored using continuous SV‐P and intermittent CI‐Li at three predefined time points: (1) supine baseline after anesthetic induction, (2) after head‐up tilt (HUT), and (3) during phenylephrine infusion in HUT. Agreement in SVI changes was assessed by directional concordance, absolute changes and Bland–Altman analysis, and the impact of vascular load was further explored by examining SVRI. A ≥ 10% change in SVI was defined as clinically relevant.

**Results:**

A total of 19 patients were included. Directional concordance between the two methods was 100% in both interventions. The SV‐P registered SVI changes ≥ 10% in all patients during both interventions, while lithium dilution registered this in 16 and 18 patients, respectively. The SV‐P consistently overestimated SVI changes compared with CI‐Li, with a non‐significant mean difference of −1.6 mL/m^2^ (95% CI: −4.2 to 1.1, *p* = 0.28) from baseline to HUT, and a significant overestimation during phenylephrine infusion with a mean difference of 4.9 mL/m^2^ (95% CI: 2.5 to 7.4, *p* = 0.001). Relative discrepancies between SV‐P and CI‐Li appeared to increase with higher SVRI.

**Conclusion:**

The arterial pulse wave‐based algorithm detected clinically relevant changes in SVI with 100% sensitivity and showed a 100% concordance with lithium dilution in the direction of change. However, it generally overestimated absolute SVI changes, and most markedly so during phenylephrine administration. Relative discrepancies in absolute SVI values appeared to increase with higher SVRI, highlighting that vascular load affects agreement.

**Editorial Comment:**

This study compares the performance of one device's pulse contour‐based estimates for stroke volume to estimates of stroke volume, same device, based on dilution‐based cardiac output and observed heart rate. This was also tracked during anesthesia interventions expected to alter peripheral vascular tone. These 2 methods for stroke volume (index) estimation agreed in direction of change with the clinical interventions, while pulse contour analysis appeared to overestimate changes in stroke volume compared to the flow‐based estimates.

## 1 Introduction

Vasoconstriction is commonly used for hemodynamic support during anesthesia where it can induce changes in preload and thereby stroke volume (SV) [1]. Accurate monitoring of SV and cardiac output (CO) is required to fully understand these hemodynamic changes and to guide decision making in fluid management and vasoactive therapy [2, 3, 4]. Gold standard methods such as pulmonary artery catheterization (PAC) with thermodilution or lithium dilution provide reliable measurements [5, 6], but their clinical use is limited because they are invasive and only provide intermittent measurements [7, 8]. Consequently, less invasive monitoring techniques are increasingly used. These include algorithmic systems that offer continuous estimation of CO and SV based on pulse contour analysis.

Pulse contour algorithms generally model the SV from mathematical calculations based on arterial pulse wave analysis while incorporating patient specific parameters and assumptions about arterial compliance, elastance, and systemic vascular resistance (SVR) [9]. There are many systems using pulse contour analysis, each with different algorithms both with and without calibration, but it is important to note that calibration does not improve the ability to detect dynamic changes in any system [10]. Furthermore, the validity of these underlying assumptions may be challenged during pronounced alterations in vascular tone. While the accuracy of pulse contour analysis has been shown to deteriorate in states of low SVR [11, 12], less attention has been given toward conditions characterized by increasing SVR, as commonly observed during vasopressor administration.

Vasopressor induced increases in SVR may alter arterial compliance and wave reflection, compromising the accuracy of stroke volume estimates derived from pulse contour analysis. Consequently, absolute levels of SVR may influence agreement with reference methods.

In this study, we aimed to evaluate the ability of the pulse contour analysis based PulseCO (LiDCO, London, UK) to detect clinically relevant changes in SV index (SVI) during manipulation of vascular tone. Furthermore, we sought to assess the impact of absolute SVR index (SVRI) levels on the agreement between pulse contour derived SVI and lithium dilution as a gold standard method.

## 2 Methods

### 2.1 Study Design and Participants

This was a pre‐planned secondary analysis of a prospective observational study evaluating hemodynamic effects of vasoconstriction on SVI [1]. A total of 19 of the 20 patients included in the present study were also included in our previously published study based on the same experimental protocol^1^. The present study represents a pre‐planned secondary analysis of these data and addresses a distinct research question. The study was conducted at a large university hospital and included patients scheduled for gastrointestinal surgery under general anesthesia. When the investigators were available, patients were included the day before surgery, where both oral and written informed consent were obtained. Patient with a history of heart failure, ischemic heart disease, atrial fibrillation, or cerebrovascular disease were excluded [1].

In the supine position, total intravenous general anesthesia was induced according to local guidelines [1]. A radial artery catheter was inserted and connected to a LiDCO Unity monitor (LiDCO, London, UK) for continuous hemodynamic monitoring via arterial pulse wave analysis using the PulseCO algorithm (SV‐P). This system provided real‐time measurements of SVRI, mean arterial pressure (MAP), heart rate (HR), SVI, and cardiac index (CI). The data were recorded and later analyzed using LiDCOviewPRO V1 (LiDCO, London, UK). In addition to continuous monitoring, CI was also assessed using lithium dilution with the LiDCO method (CI‐Li) at specific timepoints. A lithium‐sensitive electrode was inserted into the arterial line, and a roller pump was used to draw arterial blood at a steady rate, generating a lithium concentration‐time curve. CO was then calculated using the Stewart‐Hamilton equation following the administration of 0.3 mmol lithium through a central line or a suitable peripheral vein (external jugular, basilic, or brachial) [13, 14], and CI was calculated by dividing CO by body surface area.

After induction, SV‐P derived parameters were measured continuously and only interrupted during CI‐Li measurements. CI‐Li measurements were obtained at three time points: (1) baseline, (2) after positioning the patient in the head‐up tilt (HUT) position, and (3) in HUT position after steady state with phenylephrine infusion had been reached (see below).

The baseline measurements were obtained once SV‐P derived SVI, CI and MAP values had stabilized. The operating table was then adjusted to a 25‐degree HUT position, and after a new steady state was reached, the second CI‐Li measurement was taken. Finally, while the patient remained in HUT, a phenylephrine infusion (15−30μg/min) was administered to achieve a new steady state with stroke volume variation (SVV) below 12%, at which the final CI‐Li measurement was performed. Each lithium derived measurement period included 2 CI recordings, with a third measurement performed if the initial two differed by more than 15%.

### 2.2 Data Endpoints

The primary endpoint for analysis was SVI, which was derived from the CI‐Li measurements at specific timepoints and measured continuously using the PulseCO algorithm.

The CI‐Li measurements necessitated temporary disconnection of the signal from the PulseCO algorithm. Therefore, we used the mean SVI derived from PulseCO for 15 s before and after each CI‐Li measurement for the comparisons. Since the lithium derived measurements provided only a CI value, we used the mean HR from the 15 s before and after each measurement to calculate CI‐Li derived SVI.

### 2.3 Statistical Analysis

Data distribution was previously assessed using the Kolmogorov–Smirnov test and qq‐plots [1]. During this study, we additionally assessed the distribution visually using histograms and qq‐plots for the variables presented in Table 1 and SVI. Data throughout this article are presented as means with standard deviations (SD) or medians with interquartile range (IQR).

To evaluate the level of directional agreement between paired measurements, we analyzed concordance, defined as the fraction of events in which the two measurement methods showed the same direction of change in SVI. To evaluate the PulseCO algorithm's ability to detect clinically relevant changes, we compared relative SVI changes for each intervention and defined a difference greater than 10% as clinically relevant.

Agreement in absolute SVI changes was evaluated by comparing the absolute changes produced by each intervention for both methods using a paired *t*‐test. The comparison was reported with the corresponding *p* value and 95% confidence interval (CI) for the mean difference between the methods.

Bland–Altman analysis [15] was performed for paired values measured by two separate methods, focusing on changes in SVI rather than absolute SVI values. The mean difference in SVI changes between the two methods was termed “bias”. The upper and lower limits of agreement were calculated as the bias ±1.96 SD of the paired differences. The percentage error was calculated according to the method proposed by Critchley and Critchley as 1.96·SD of the bias divided by the mean SVI of the paired measurements [15, 16].

To evaluate potential systematic inaccuracies in SVI estimates, we assessed the relative discrepancies between absolute SVI values obtained from the two methods. For absolute SVI values only, using data obtained during phenylephrine administration, we examined how absolute SVRI and MAP levels influenced the accuracy of SV‐P derived SVI. Relative discrepancies were calculated as the difference between SV‐P derived SVI and CI‐Li derived SVI, divided by CI‐Li derived SVI, and were plotted against SVRI and MAP to explore whether increasing vascular load was associated with larger relative disagreement.

No formal sample size calculation was performed for this secondary analysis, as the study size was determined by the original study design.

Statistical analyses were performed using R version 4.3.3 (R Core Team, 2024; R Foundation for Statistical Computing, Vienna, Austria) as well as R Studio version 2024.12.1 + 563 (Posit team, 2024) and Excel Version 16.95.1 (25031528).

### 2.4 Ethics

The study was initiated by the Department of Anesthesia, Hvidovre University Hospital. It was approved by the regional ethics committee (H‐21032981) and registered at ClinicalTrials.gov (NCT05193097). Processing of personal data was authorized by the Danish Data Protection Agency (AHH‐2016‐095).

## 3 Results

A total of 21 patients were enrolled. One patient was excluded on the day of surgery due to equipment failure, and another was omitted from the analysis because of missing algorithm data. Consequently, the statistical analysis included data from 19 patients. Patient characteristics are reported in Table 1.

**TABLE 1:** Patient characteristics.

Patients [*n*]	19
Mean age [years (SD)]	58 (15)
Sex, female [*n* (%)]	10 (53)
Mean height [cm (SD)]	172 (8.5)
Mean weight [kg (SD)]	83 (17)
Mean BSA [$m^{2}$ (SD)]	1.95 (0.2)
ASA [*n*]	
1	4
2	12
3	3
4	0
Type of surgery [*n*]	
Upper GI	10
Lower GI	7
Hernia repair	2

Abbreviations: ASA, American Society of Anesthesiologists; BSA, body surface area; GI, gastrointestinal; SD, standard deviation.

Hemodynamic variables at baseline, during head‐up tilt (HUT), and during phenylephrine administration are presented in Table 2. Compared with baseline, HUT was associated with lower MAP, CI, and SVI values. During phenylephrine administration, MAP and SVRI increased, and SVI and CI values were higher than during HUT. Absolute values of CI and SVI are shown for both methods (SV‐P and CI‐Li).

**TABLE 2:** Hemodynamic characteristics at baseline, during head‐up tilt (HUT), and during phenylephrine administration.

Hemodynamic characteristics	Baseline	HUT	Phenylephrine	*p*
MAP (mmHg)	61.0 (54.5:67.5)	40.0 (37.0:41.5)	85.0 (78.0:92.0)	< 0.001
SVRI (dyn·s·cm^−5^·m^2^)	485 (439:707)	438 (339:666)	684 (590:879)	< 0.001
HR (bpm)	60.4 (55.0:66.8)	60.5 (55.8:67.3)	52.0 (47.1:58.7)	< 0.001
CI‐P (L·min^−1^·m^−2^)	2.1 (1.8:2.5)	1.6 (1.3:1.9)	2.3 (2.0:2.4)	< 0.001
SVI‐P (mL·m^−2^)	33.3 (32.2:40.7)	24.4 (22.0:28.9)	44.2 (41.8:47.0)	< 0.001
CI‐Li (L·min^−1^·m^−2^)	2.6 (2.4:2.9)	2.1 (1.9:2.4)	2.5 (2.1:2.7)	< 0.001
SVI‐Li (mL·m^−2^)	40.9 (37.0:49.0)	34.9 (28.7:39.8)	46.7 (40.4:57.1)	< 0.001

*Note:* Data are reported as median (interquartile range). *p* values reflect overall differences across the three time points.

Abbreviations: CI‐Li, cardiac index derived from lithium dilution; CI‐P, cardiac index derived from the PulseCO algorithm; HR, heart rate; MAP, mean arterial pressure; SVI‐Li, stroke volume index derived from lithium dilution; SVI‐P, stroke volume index derived from the PulseCO algorithm; SVRI, systemic vascular resistance index.

The directional change between the paired SVI measurements before and after each intervention showed 100% concordance from baseline to HUT and 100% concordance from HUT to HUT + phenylephrine infusion. Relative changes in SVI are presented in Table 3. The bold highlights are cases where a change of less than 10% was measured. When moving from baseline to HUT, both methods detected a change > 10% in 16/19 patients, whereas only SV‐P detected a clinically relevant change in the remaining 3 patients. During phenylephrine administration, 18/19 patients showed a clinically relevant change with both methods, whereas only SV‐P did so in the remaining patient.

**TABLE 3:** Relative changes in stroke volume index (SVI).

PT	BL → HUT SVI‐Li (%)	BL → HUT SVI‐P (%)	HUT → PE SVI‐Li (%)	HUT → PE SVI‐P (%)
1	−13.8	−12.3	35.8	82.3
2	−15.8	−30.3	32.6	97.2
3	−11.7	−26.6	30.9	40.4
4	−14.2	−29.8	45.8	88.6
5	−12.9	−29.9	69.4	77.4
6	−18.5	−34.0	34.9	89.0
7	−12.9	−21.4	48.2	69.7
8	−30.3	−36.7	67.5	124.9
9	−26.7	−27.4	45.7	43.1
10	**−24.0**	−48.4	49.0	183.8
11	**−6.0** ^a^	−26.4	23.9	102.8
12	**−8.6** ^a^	−20.5	17.9	39.9
13	−25.5	−21.8	**0.5** ^a^	33.5
14	−41.3	−26.2	16.5	34.9
15	−33.8	−35.0	22.3	27.6
16	−15.1	−31.9	38.8	92.5
17	**−8.5** ^a^	−28.3	52.7	131.5
19	−18.1	−31.6	63.1	100.3
20	−33.8	−28.5	41.2	47.6

Abbreviations: BL, baseline; HUT, head up tilt; PE, phenylephrine; SVI‐Li, SVI derived from lithium dilution. SVI‐P, SVI derived from the PulseCO algorithm.

^a^ Cases where a change of less than 10% was measured.

Table 4 reports mean changes in SVI for each intervention measured by the two methods, along with the mean difference between them. There was a high level of agreement from baseline to HUT where the difference was non‐significant, but the SV‐P overestimated SVI change from HUT to HUT + phenylephrine by 4.9 mL/m^2^, which was statistically significant (*p* = 0.001).

**TABLE 4:** Changes in stroke volume index.

Intervention	Mean ∆SVI (mL/m^2^) lithium (SD)	Mean ∆SVI (mL/m^2^) algorithm (SD)	Mean difference (mL/m^2^) (95% CI)	*p*
Baseline → HUT	−8.9 (6.2)	−10.5 (3.7)	−1.6 (−4.2; 1.1)	0.28
HUT → HUT+PE	13.5 (6.6)	18.4 (5.8)	4.9 (2.5; 7.4)^a^	0.001

Abbreviations: CI, confidence interval; HUT, head‐up tilt; PE, phenylephrine; SD, standard deviation; SVI, stroke volume index.

^a^ Results with statistical significance.

Figures 1 and 2 show the Bland–Altman plots for the two interventions, confirming mean ΔSVI biases of −1.6 mL/m^2^ (baseline to HUT) and 4.9 mL/m^2^ (HUT to HUT + phenylephrine) between SV‐P and CI‐Li derived SVI, respectively. Limits of agreement (LoA), calculated as bias ±1.96 SD, were ±11.6 and ±10.6 mL/m^2^, respectively. The percentage error for the paired methods for each intervention was 119% and 67%, respectively.

**FIGURE 1:**
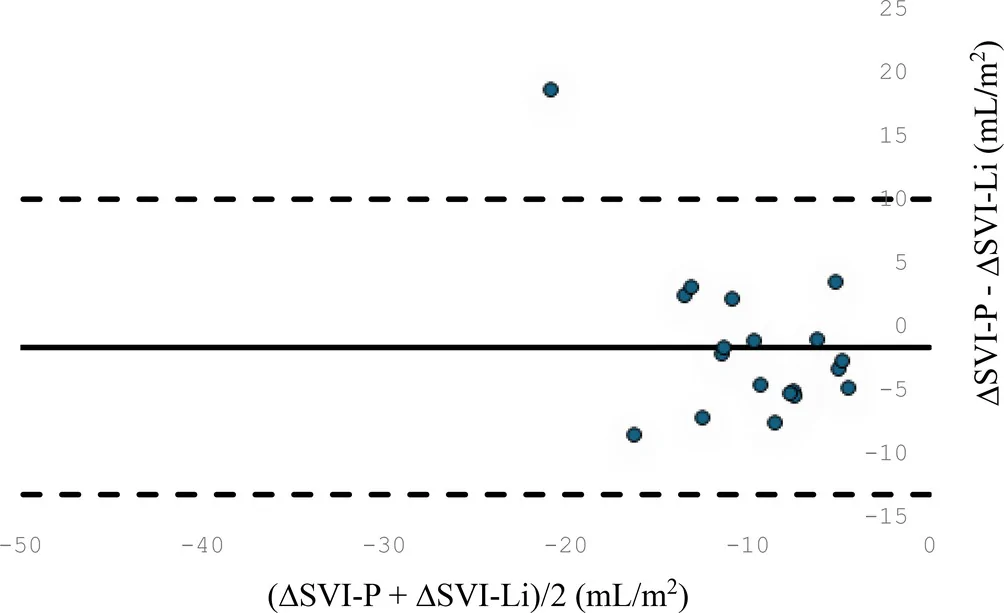
Bland–Altman analysis for the paired methods´ ∆SVI. Baseline → HUT. Bland–Altman plot of SVI changes between the two methods. The *x*‐axis shows the mean change in SVI, and the *y*‐axis shows the difference in SVI changes between methods. Solid line = Bias (mean difference). Dashed lines = Limit of agreement (Bias ±1.96 standard deviation (SD)). The bias was −1.6 mL/m^2^ (95% CI −4.2 to 1.1). The upper limit of agreement was 10.1 mL/m^2^ (95% CI 5.5 to 14.7), and the lower limit of agreement was −13.2 mL/m^2^ (95% CI −17.8 to −8.6) SVI = Stroke Volume Index. ∆ SVI‐P = ∆ SVI derived from the PulseCO algorithm. ∆ SVI‐Li = ∆ SVI derived from lithium dilution.

**FIGURE 2:**
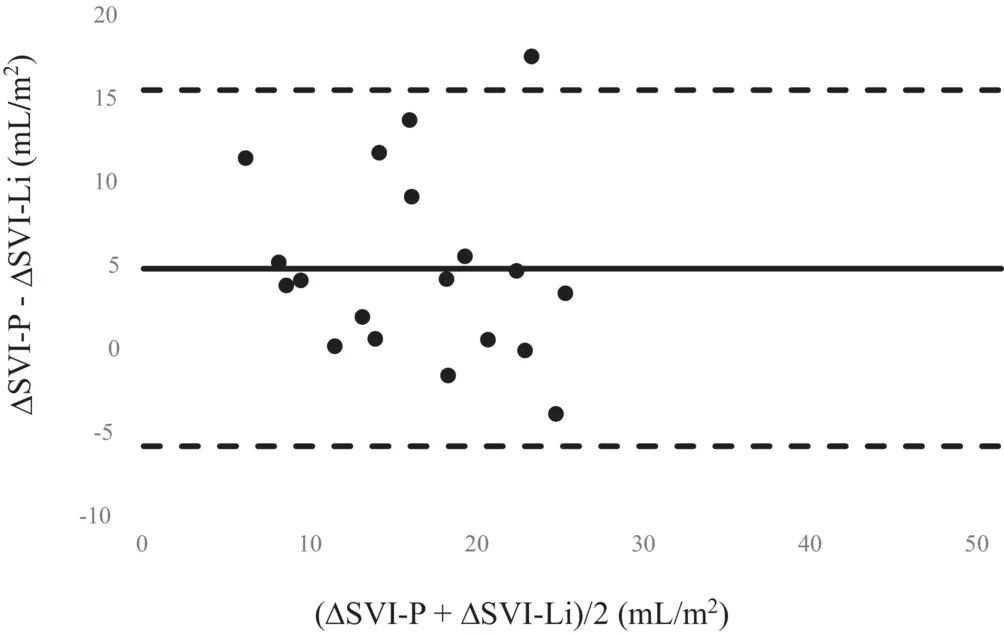
Bland–Altman analysis for the paired methods´ ∆SVI. HUT → HUT + Phenylephrine. Bland–Altman plot of SVI changes between the two methods. The *x*‐axis shows the mean change in SVI, and the y‐axis shows the difference in SVI changes between methods. Solid line = Bias (mean difference). Dashed lines = Limit of agreement (Bias ±1.96 standard deviation (SD)). The bias was 4.9 mL/m^2^ (95% CI 2.5 to 7.4). The upper limit of agreement was 15.5 mL/m^2^ (95% CI 11.4 to 19.8), and the lower limit of agreement was −5.7 mL/m^2^ (95% CI −10.0 to −1.5). SVI = Stroke Volume Index. ∆SVI‐P = ∆SVI derived from the PulseCO algorithm. ∆SVI‐Li = ∆SVI derived from lithium dilution.

Relative discrepancies between absolute SVI values obtained by the two methods are shown in Figures 3 and 4. When plotted against SVRI, relative differences increased with higher SVRI values (Figure 3), demonstrating a moderate positive association. A weaker positive association was observed between relative differences and MAP (Figure 4). Both analyses were restricted to measurements obtained during phenylephrine administration. Correlation analysis demonstrated a moderate association between relative differences and SVRI (*r* = 0.47, 95% CI 0.02–0.76, *p* = 0.041), whereas the association with MAP was weaker and not statistically significant (*r* = 0.32, 95% CI −0.16 to 0.68, *p* = 0.180). Sensitivity analysis excluding the observation with an SVRI value of approximately 2000 yielded similar results (Figure S1; *r* = 0.52, 95% CI 0.06–0.79, *p* = 0.029).

**FIGURE 3:**
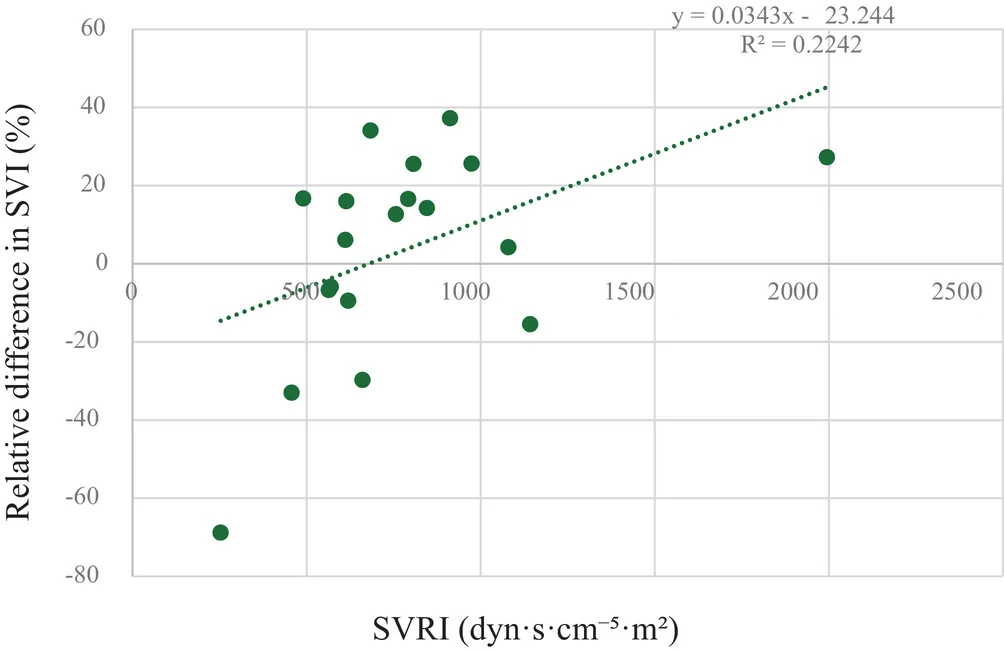
Relationship between relative difference in SVI and SVRI after phenylephrine administration. Relative discrepancies between absolute stroke volume index (SVI) values obtained by the pulse contour analysis based PulseCO system and the lithium dilution method plotted against systemic vascular resistance index (SVRI) using post intervention data after phenylephrine administration. Pearson's correlation coefficient (*r*) = 0.47 (95% CI 0.02–0.76), *p* = 0.041.

**FIGURE 4:**
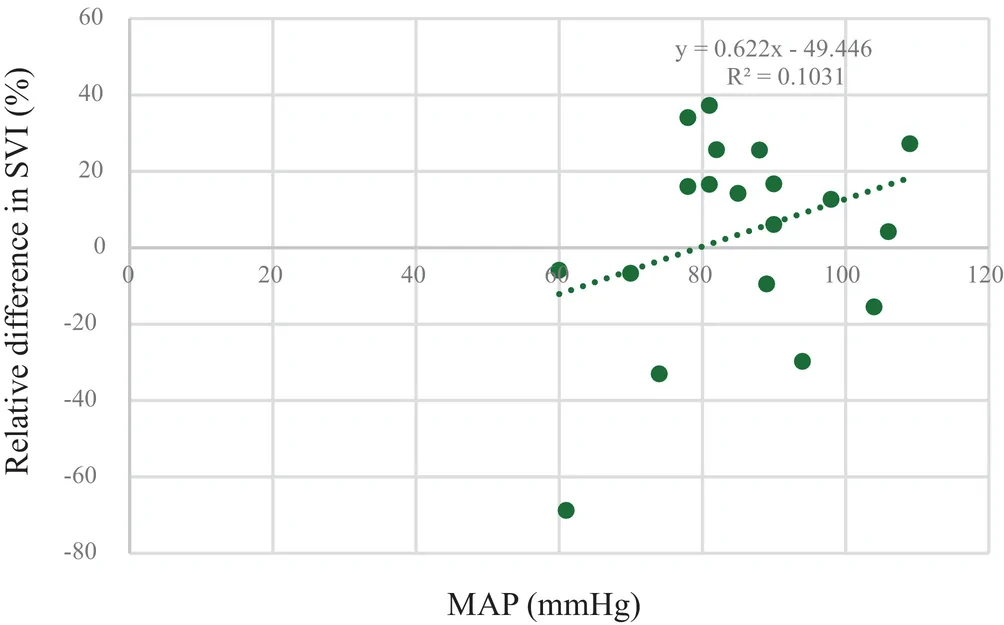
Relationship between relative difference in SVI and MAP after phenylephrine administration. Relative discrepancies between absolute stroke volume index (SVI) values obtained by the pulse contour analysis based PulseCO system and the lithium dilution method plotted against mean arterial pressure (MAP) using post intervention data after phenylephrine administration. Pearson's correlation coefficient (*r*) = 0.32 (95% CI −0.16 to 0.68), *p* = 0.180.

## 4 Discussion

In this observational study of 19 subjects, we evaluated pulse contour analysis‐derived measurements of SVI changes induced by alterations of vascular resistance, using lithium dilution as a gold standard comparator.

Our directional concordance analysis showed that the two methods consistently described SVI changes in the same direction. It is essential that SVI monitoring can detect changes of a magnitude that is considered clinically relevant, which is commonly defined as ≥ 10% [17]. In our study, the SV‐P method detected all the intervention‐related SVI changes that were clinically relevant according to the CI‐Li reference, but also identified a few more changes as clinically relevant. This indicates that the algorithm demonstrated 100% sensitivity to clinically relevant changes in SVI but may also generate false positives by overestimating SVI. This should be considered when using SV‐P derived measurements in clinical practice.

### 4.1 Absolute SVI Changes

SV‐P derived measurements consistently reported larger absolute changes in SVI compared with CI‐Li in both interventions (Table 4). With phenylephrine administration, this difference was both statistically significant and clinically relevant, with the SV‐P derived measurement reporting significantly greater changes in SVI. This may reflect a known limitation of pulse contour analysis systems which are sensitive to changes in vascular tone, especially under the administration of vasopressors [18, 19, 20]. However, the present findings should be interpreted within the physiological context studied. Although the observed overestimation of ΔSVI was most pronounced during phenylephrine administration, PulseCO also overestimated ΔSVI during Baseline→HUT transitions, suggesting that state‐dependent bias across changing hemodynamic conditions may have contributed to the observed discrepancies. Monnet et al. thus similarly showed that FloTrac (Edwards Lifesciences, Irvine, CA, USA) systematically overestimates CI during norepinephrine administration due to change in vascular tone in a setup where there was no significant change in SVI [18]. Although the FloTrac algorithm differs from the PulseCO, and another vasopressor was used, this study also clearly illustrates the well‐known limitation described above.

The Bland–Altman analysis supported these findings, showing a mean bias of 4.9 mL/m^2^ (HUT → HUT + phenylephrine), but with wide LoA from −5.7 to 15.5 mL/m^2^. Thus, although a statistically significant difference between the two methods was observed, with SV‐P systematically giving higher estimates than CI‐Li with 95% confidence, the wide LoA shows that individual measurements can vary. Therefore, the results should be interpreted with caution on an individual basis. The large percentage error of 67% further indicates poor agreement and insufficient precision.

Similar challenges with variability have previously been reported with the uncalibrated PulseCO algorithm. In a study by Phan et al., the percentage error reached 54% despite minimal bias, indicating substantial individual variability [21]. Broch et al. reported even greater inaccuracy after induction, with an error of 86%, which only improved to acceptable levels after calibration [22]. Several other studies argue that the most accurate estimates are obtained through calibrated pulse contour analysis methods [23, 24]. However, it should be noted that these studies have primarily focused on absolute values, where calibration can be highly beneficial, as it aligns the measurements with an established reference method such as lithium dilution. In contrast, our study primarily examines dynamic changes. In this context, calibration cannot influence the results, as the correction factor applied during calibration remains constant, and the estimation of changes is determined entirely by the algorithm (regardless of calibration). Assessing such changes through calibration would require recalibration before and after each intervention, effectively relying on the calibration itself rather than the algorithm. This underscores that, when assessing absolute changes, continuous and reliable SVI/CI monitoring without frequent recalibration is essential [25].

### 4.2 Influence of Vascular Load

To explore whether vascular load influenced agreement between the two methods, we examined relative disagreement between the two methods based on absolute SVI values. When relative discrepancies between SV‐P derived and CI‐Li derived absolute SVI values were plotted against SVRI (Figure 3), higher SVRI values were associated with larger positive relative disagreement. A similar pattern was observed when relative discrepancies in absolute SVI were plotted against MAP (Figure 4). Both analyses were restricted to measurements obtained after phenylephrine administration. These findings suggest that agreement between the two methods varied across different levels of vascular load, even when absolute SVI values, rather than intervention induced changes, were considered. However, these analyses were exploratory and hypothesis‐generating. Although a moderate association was observed between relative differences and SVRI, confidence intervals remained relatively wide, and the study was not powered to robustly evaluate these relationships. The findings should therefore be interpreted with caution.

These observations are consistent with previous studies demonstrating that systemic vascular resistance is a key determinant of the accuracy of pulse contour derived estimates. Prior studies conducted in vasodilated states, including during vasodilator administration, have reported that arterial pulse contour analysis tends to underestimate SV under conditions of low SVR and show large absolute differences [11, 12].

Our results indicate that under conditions of increased SVRI and elevated arterial pressure, such as during vasopressor administration, absolute SV‐P derived measurements may show increasing disagreement with CI‐Li derived measurements and a tendency towards overestimation of SVI. The present findings should be interpreted in the context of the studied physiological conditions. Although phenylephrine increased SVRI, the intervention was applied in anesthetized patients who were rendered preload responsive by the study design. Thus, the observed increase in vascular resistance represented a relative increase from a predominantly vasodilated state rather than a transition to a markedly elevated SVRI state. Nevertheless, this remains a clinically relevant scenario, as vasopressors are frequently administered in preload‐responsive patients during anesthesia. Whether similar effects would be observed in patients with substantially higher SVRI warrants further investigation.

### 4.3 Limitations

The present study has several limitations. No formal sample size calculation was performed for this secondary analysis, as the sample size was determined by the primary study. The relatively small sample size limits the statistical power and increases the uncertainty of the estimated effects. Although several predefined analyses demonstrated statistically significant findings, smaller effects may have gone undetected. Hence, the findings should be interpreted with caution, particularly with respect to their generalizability to a broader population. Notably, hemodynamic status was partially manipulated as all patients were rendered preload dependent by the study design. Furthermore, although phenylephrine increased SVRI, the intervention represented a relative increase from a predominantly vasodilated baseline state rather than a transition to markedly elevated SVRI levels. Therefore, the findings should primarily be interpreted within the physiological conditions investigated in the present study. Moreover, the participants had few comorbidities that could potentially influence the SVI estimates, which further limits the generalizability of our findings. In this study, SVI increased in all participants in response to vasoconstriction due to preload dependency. Consequently, the present findings do not reflect how the PulseCO algorithm performs during vasoconstriction in the absence of preload dependency.

Another limitation relates to the assumption of hemodynamic stability during lithium dilution measurements. However, all measurements were reviewed during data processing, and the 15‐s periods before and after lithium dilution were considered hemodynamically stable with only minor variation.

## 5 Conclusion

During the two interventions designed to manipulate preload and thus SVI/CI in anesthetized patients, the pulse contour analysis based PulseCO detected clinically relevant SVI changes with 100% sensitivity. While showing complete directional concordance with lithium dilution, the PulseCO algorithm showed individual variability and tended to overestimate the absolute SVI changes, especially and significantly with vasopressor administration. When considering absolute SVI values, agreement between the two methods varied with vascular load, with exploratory analyses suggesting greater discrepancies at higher SVRI and MAP values. Clinicians should therefore exercise caution when interpreting changes in algorithmic‐derived SVI during vasopressor administration, as the two methods are not interchangeable—particularly when precise assessment of SVI, rather than directional changes, is required.

## Author Contributions

Jonathan Winther Olsen, Henrik Wolsted, and Nicolai Bang Foss developed the research question and methodology for the present secondary analysis. Jakob Højlund and Mirjana Cihoric contributed to the acquisition of data. Jonathan Winther Olsen performed the analyses and wrote the first draft of the manuscript. All authors contributed to the interpretation of the results, critically revised the manuscript, and approved the final manuscript.

## Funding

The authors have nothing to report.

## Conflicts of Interest

Nicolai Bang Foss has received speaker's fees from Masimo. The remaining authors declare no conflicts of interest.

## Supporting information


**Figure S1:** Relationship between relative difference in SVI and SVRI after phenylephrine administration excluding the observation with SVRI≈2000. Relative discrepancies between absolute stroke volume index (SVI) values obtained by the PulseCO system and the lithium dilution method plotted against systemic vascular resistance index (SVRI) using measurements obtained after phenylephrine administration. The observation with an SVRI value of approximately 2000 was excluded as part of a sensitivity analysis.

## Data Availability

The data that support the findings of this study are available from the corresponding author upon reasonable request. The data are not publicly available due to privacy and ethical restrictions.

## References

[1] J. Højlund , M. Cihoric , and N. B. Foss , “Vasoconstriction With Phenylephrine Increases Cardiac Output in Preload Dependent Patients,” Journal of Clinical Monitoring and Computing 38 (2024): 997–1002. 10.1007/s10877-024-01186-7 PMC11427527 38907106

[2] S. M. Abbas and A. G. Hill , “Systematic Review of the Literature for the Use of Oesophageal Doppler Monitor for Fluid Replacement in Major Abdominal Surgery,” Anaesthesia 63 (2008): 44–51. 10.1111/j.1365-2044.2007.05233.x 18086070

[3] M. Bundgaard‐Nielsen , K. Holte , N. H. Secher , and H. Kehlet , “Monitoring of Peri‐Operative Fluid Administration by Individualized Goal‐Directed Therapy: Review Article,” Acta Anaesthesiologica Scandinavica 51 (2007): 331–340. 10.1111/j.1399-6576.2006.01221.x 17390421

[4] D. J. Funk , E. W. Moretti , and T. J. Gan , “Minimally Invasive Cardiac Output Monitoring in the Perioperative Setting,” Anesthesia and Analgesia 108 (2009): 887–897. 10.1213/ane.0b013e31818ffd99 19224798

[5] M. G. Costa , G. Della Rocca , P. Chiarandini , et al., “Continuous and Intermittent Cardiac Output Measurement in Hyperdynamic Conditions: Pulmonary Artery Catheter vs. Lithium Dilution Technique,” Intensive Care Medicine 34 (2008): 257–263. 10.1007/s00134-007-0878-6 17922106

[6] A. S. Rali , A. Butcher , R. J. Tedford , et al., “Contemporary Review of Hemodynamic Monitoring in the Critical Care Setting,” US Cardiology Review 16 (2022): e12. 10.15420/usc.2021.34 PMC11588176 39600839

[7] D. A. Reuter , C. Huang , T. Edrich , S. K. Shernan , and H. K. Eltzschig , “Cardiac Output Monitoring Using Indicator‐Dilution Techniques: Basics, Limits, and Perspectives,” Anesthesia and Analgesia 110 (2010): 799–811. 10.1213/ANE.0b013e3181cc885a 20185659

[8] D. C. Evans , V. A. Doraiswamy , M. P. Prosciak , et al., “Complications Associated With Pulmonary Artery Catheters: A Comprehensive Clinical Review,” Scandinavian Journal of Surgery 98 (2009): 199–208. 10.1177/145749690909800402 20218415

[9] J. Grensemann , “Cardiac Output Monitoring by Pulse Contour Analysis, the Technical Basics of Less‐Invasive Techniques,” Frontiers in Medicine (Lausanne) 5 (2018): 64, 10.3389/fmed.2018.00064. PMC5845549 29560351

[10] J. Bernards , M. Mekeirele , B. Hoffmann , and Y. Peeters , “Hemodynamic Monitoring: To Calibrate or Not to Calibrate? Part 2‐Non‐Calibrated Techniques,” Anestezjologia Intensywna Terapia 47 (2015): 517–532.

[11] K. Yamashita , T. Nishiyama , T. Yokoyama , H. Abe , and M. Manabe , “Effects of Vasodilation on Cardiac Output Measured by PulseCO,” Journal of Clinical Monitoring and Computing 21 (2007): 335–339. 10.1007/s10877-007-9093-9 17896183

[12] M. Biais , E. Mazocky , L. Stecken , et al., “Impact of Systemic Vascular Resistance on the Accuracy of the Pulsioflex Device,” Anesthesia and Analgesia 124 (2017): 487–493. 10.1213/ANE.0000000000001591 28067706

[13] C. Garcia‐Rodriguez , J. Pittman , C. H. Cassell , et al., “Lithium Dilution Cardiac Output Measurement: A Clinical Assessment of Central Venous and Peripheral Venous Indicator Injection,” Critical Care Medicine 30 (2002): 2199–2204. 10.1097/00003246-200210000-00004 12394944

[14] J. W. Moore , J. M. Kinsman , W. F. Hamilton , and R. G. Spurling , “Studies on the Circulation,” American Journal of Physiology 89 (1929): 331–339, 10.1152/ajplegacy.1929.89.2.331.

[15] J. Martin Bland and D. G. Altman , “Statistical Methods for Assessing Agreement Between Two Methods of Clinical Measurement,” Lancet 327 (1986): 307–310. 2868172

[16] L. A. H. Critchley and J. A. J. H. Critchley , “A Meta‐Analysis of Studies Using Bias and Precision Statistics to Compare Cardiac Output Measurement Techniques,” Journal of Clinical Monitoring and Computing 15 (1999): 85–91. 10.1023/a:1009982611386 12578081

[17] R. Barthélémy , M. Kindermans , P. Delval , et al., “Accuracy of Cumulative Volumes of Fluid Challenge to Assess Fluid Responsiveness in Critically Ill Patients With Acute Circulatory Failure: A Pharmacodynamic Approach,” British Journal of Anaesthesia 128 (2022): 236–243. 10.1016/j.bja.2021.10.049 34895718

[18] X. Monnet , N. Anguel , M. Jozwiak , C. Richard , and J. L. Teboul , “Third‐Generation FloTrac/Vigileo Does Not Reliably Track Changes in Cardiac Output Induced by Norepinephrine in Critically Ill Patients,” British Journal of Anaesthesia 108 (2012): 615–622. 10.1093/bja/aer491 22265900

[19] P. S. Halvorsen , A. Espinoza , R. Lundblad , et al., “Agreement Between PiCCO Pulse‐Contour Analysis, Pulmonal Artery Thermodilution and Transthoracic Thermodilution During Off‐Pump Coronary Artery By‐Pass Surgery,” Acta Anaesthesiologica Scandinavica 50 (2006): 1050–1057. 10.1111/j.1399-6576.2006.01118.x 16987335

[20] M. Hadian , H. K. Kim , D. A. Severyn , and M. R. Pinsky , “Cross‐Comparison of Cardiac Output Trending Accuracy of LiDCO, PiCCO, FloTrac and Pulmonary Artery Catheters,” Critical Care 14 (2010): R212. 10.1186/cc9335 PMC3220011 21092290

[21] T. D. Phan , R. Kluger , C. Wan , D. Wong , and A. Padayachee , “A Comparison of Three Minimally Invasive Cardiac Output Devices With Thermodilution in Elective Cardiac Surgery,” Anaesthesia and Intensive Care 39 (2011): 1014–1021. 10.1177/0310057X1103900606 22165352

[22] O. Broch , J. Renner , J. Höcker , et al., “Uncalibrated Pulse Power Analysis Fails to Reliably Measure Cardiac Output in Patients Undergoing Coronary Artery Bypass Surgery,” Critical Care 15 (2011): R76. 10.1186/cc10065 PMC3222009 21356060

[23] R. M. Pearse , K. Ikram , and J. Barry , “Equipment Review: An Appraisal of the LiDCOPlus Method of Measuring Cardiac Output,” Critical Care 8 (2004): 190. 10.1186/cc2852 PMC468899 15153237

[24] T. Schlöglhofer , H. Gilly , and H. Schima , “Semi‐Invasive Measurement of Cardiac Output Based on Pulse Contour: A Review and Analysis,” Canadian Journal of Anesthesia 61 (2014): 452–479. 10.1007/s12630-014-0135-8 24643474

[25] S. A. Esper and M. R. Pinsky , “Arterial Waveform Analysis,” Best Practice & Research. Clinical Anaesthesiology 28 (2014): 363–380. 10.1016/j.bpa.2014.08.002 25480767

