# Supplementary material for: Ability of the PulseCO Algorithm to Assess Changes in Stroke Volume Induced by Changes in Vascular Tone
Source: Acta Anaesthesiol Scand. 2026 Sep 27;70(10):e70338. doi: 10.1111/aas.70338 (PMC13616996; doi:10.1111/aas.70338)
Supplement: Supplementary file 1 [file AAS-70-0-s001.docx]

**Supplementary Figure S1: Relationship between relative difference in SVI and SVRI after phenylephrine administration excluding the observation with SVRI ≈ 2000**

*Relative discrepancies between absolute stroke volume index (SVI) values obtained by the PulseCO™ system and the lithium dilution method plotted against systemic vascular resistance index (SVRI) using measurements obtained after phenylephrine administration. The observation with an SVRI value of approximately 2000 was excluded as part of a sensitivity analysis.*

|  | PE |
| --- | --- |
| 806.533 | 25.5471 |
| 660.221 | -29.7524 |
| 613.825 | 15.9985 |
| 490.094 | 16.696 |
| 611.111 | 6.09577 |
| 973.563 | 25.6425 |
|  | 27.2394 |
| 791.667 | 16.5665 |
| 619.431 | -9.49001 |
| 1141.86 | -15.4988 |
| 1079.26 | 4.21244 |
| 845.143 | 14.2198 |
| 456.818 | -33.0305 |
| 756.087 | 12.6708 |
| 252.79 | -68.8099 |
| 563.351 | -6.72288 |
| 912 | 37.2334 |
| 683.598 | 34.0802 |
| 569.189 | -5.92427 |

Relative difference in SVI (%)

SVRI (dyn·s·cm⁻⁵·m²)

Pearson's correlation coefficient (r) = 0.52 (95% CI 0.06–0.79), p = 0.029.
